# Troubleshooting Carry-Over in the LC-MS Analysis of Biomolecules: The Case of Neuropeptide Y

**DOI:** 10.5702/massspectrometry.S0083

**Published:** 2020-02-26

**Authors:** Tohru Yamagaki, Takashi Yamazaki

**Affiliations:** 1 Suntory Institute for Bioorganic Research, Suntory Foundation for Life Sciences, 8–1–1 Seikadai, Seika-cho, Soraku-gun, Kyoto 619–0284, Japan

**Keywords:** neuropeptide, LC-MS, carry-over, quantitative analysis, NPY

## Abstract

We describe systematic troubleshooting of the carry-over of neuropeptide Y (NPY) in LC-MS analysis. The objective was to remove candidate parts of the LC-MS system that are responsible for carry over one-by-one. The findings indicate that the carry-over of NPY occurs on the column, particularly in the guard column and at the consumable seals of the sample-needle and high-pressure valves. The methodology demonstrates that it is possible to troubleshoot carry-over in an LC-MS system in a systematic and logical manner.

## INTRODUCTION

It is well known that faint fractions of analytes remain in an analytical system so-called memory effect or carry-over of analytes. Their chemical properties of these analytes such as their adsorption and viscosity cause the “carry-over.” In the case of biomolecules, it is the sticky molecules that remain in the system and subsequently hinder the correct quantitative analysis. It is therefore important to reduce the adsorption of these sticky compounds in the experimental system. In liquid chromatography-mass spectrometry (LC-MS), the carry-over is checked by measuring a blank solution such as water after a sample analysis. “Carry-over” means that analytes from the previous run remain in the LC-MS system, and that these analytes are detected in the next measurement. For a viable quantitative analysis, it is necessary to reduce carry-over in the LC-MS system to avoid over-estimating the amount of analyte. Carry-over trouble-shooting involves identifying the locations where carry-over occurs in the LC-MS system.^1)^ In this study, we describe a general and practical strategy for the troubleshooting of carry-over.

As an example, we analyzed the neuropeptide Y (NPY), a peptide neurotransmitter in the nervous system which functions in the regulation of feeding behavior and the control of blood pressure.^2–6)^ The NPY consists of 36 a. residues with C-terminal amidation.^7)^ The solution structure of NPY is comprised of a hydrophobic helical unit at the C-terminus and an unfolded N-terminal portion consisting of residues 1–13. The C-terminal helices of NPY can form dimers in solution and can bind to a cell surface membrane because of the hydrophobic α-helix unit.^8–11)^ Although NPY is soluble in aqueous solution, it is a sticky molecule that can also easily bind to surfaces due to its hydrophobicity. We thus describe a practical strategy for troubleshooting NPY carry-over in an LC-MS system.

## METHODS AND MATERIALS

The neuropeptide Y (Peptide Institute, Inc., Osaka, Japan), trypsin-digested bovine serum albumin (BSA) MS standard (carboxymethyl-modified) (NEW ENGLAND BioLabs, Inc., MA, USA) and HPLC grade acetonitrile (Nakalai tesque, Inc., Kyoto, Japan) were purchased from commercial sources. MilliQ water (Merck Millipore, MA, USA) was used for preparing all solutions. We used a matrix solution with trypsin-digested BSA at a concentration of 5 nmol/L in a 50% aqueous acetonitrile solution to avoid non-specific adsorption of analyte. NPY was dissolved in a matrix solution at a concentration of 50 μM as a NPY stock solution. The NPY stock solution was stored at −20°C. Blank solutions were water, a 50% aqueous acetonitrile solution and a 0.1 μM trypsin-digested BSA solution.

### LC-MS

A Nexera UPLC/HPLC system (Shimadzu Co., Kyoto, Japan) was used in conjunction with the MS instrument.

**Figure 1:**
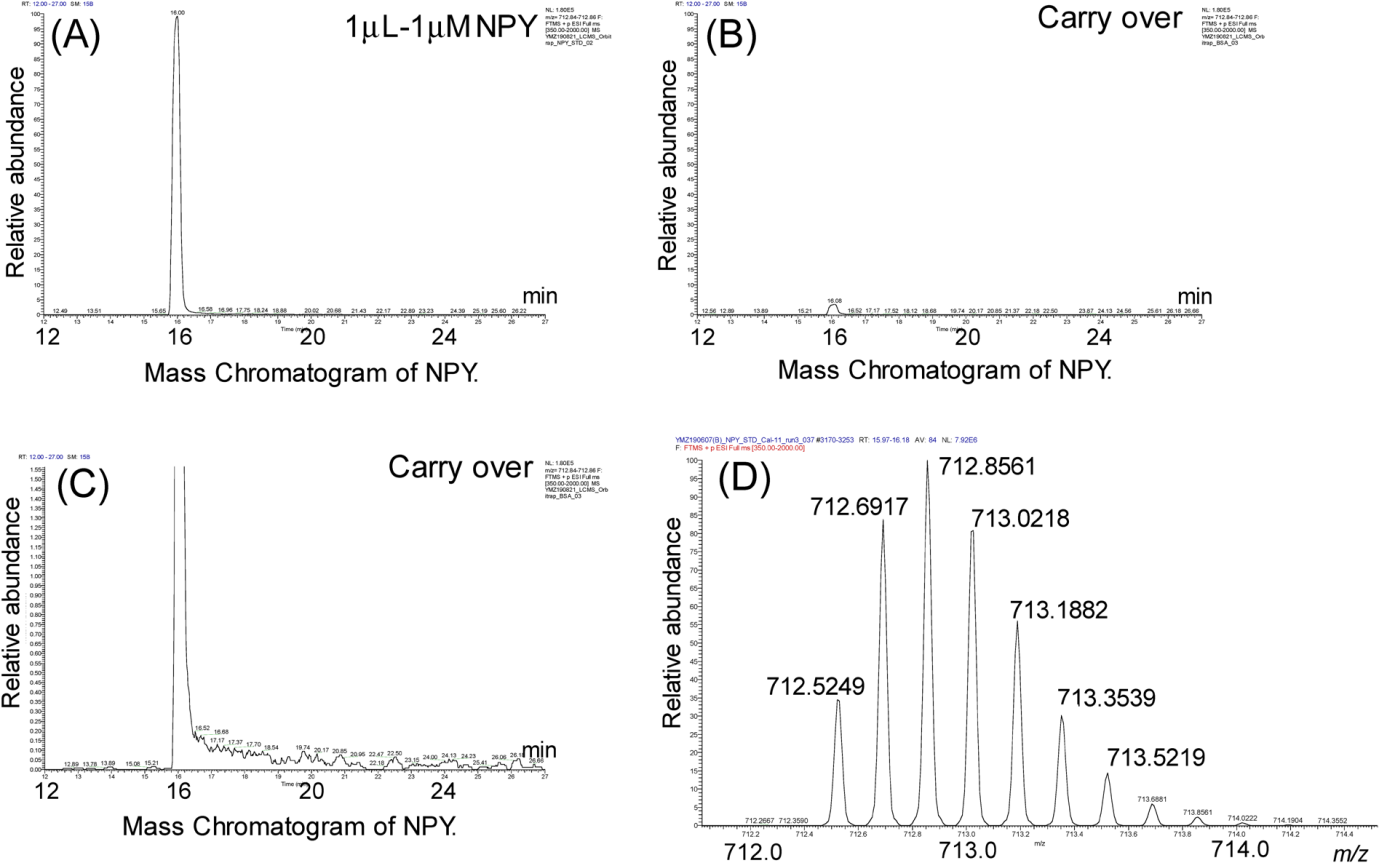
Fig. 1. The mass chromatogram and mass spectrum of NPY using LC-MS.

The columns used were an Aeris PEPTIDE XB-C18 (ID 2.1×250 mm, 2.6 μm) with a Security Guard ULTRA (Phenomenex Inc., CA, USA) guard column and a COSMOSIL 5C18-AR-II (ID 2.0×150 mm, 5 μm) (Nakalai tesque, Inc., Kyoto, Japan). The gradient elution solutions used were 0.1% formic acid/water (v/v) (solution A) and 0.1% formic acid/acetonitrile (v/v) (solution B). The gradient programs for elution in Figs. 1 and 3 were respectively; (1) 40 min-gradient program: 0–27 min (10–50% solution B), 27–28 min (50–70% solution B), 28–30 min (70% solution B), 30–31 min (70–10% solution B), 31–40 min (10% solution B); (2) 20 min gradient program: 0–7 min (10–40% solution B), 7–8 min (40–80% solution B), 8–13 min (80% solution B), 13–14 min (80–10% solution B), 14–20 min (10% solution B). To estimate carry-over, the two gradient programs were applied and other programs with minor changes were also used. The gradient programs had no effect on carry-over. The solution used to wash the sample-needle was a 50% aqueous acetonitrile solution. All MS data were acquired on a Fourier transform (FT) orbitrap linear ion-trap hybrid (FT-Orbitrap Elite) MS instrument (Thermo Fisher Scientific, Inc., MA, USA). The experimental parameters were as follows; ion-source was ESI, positive-ion mode detection, the MS resolution was 120,000 with *m*/*z* 350–2,000 mass range, capillary temperature was set to 250°C, source heater temperature was set to 500°C, flow rate of sheath gas was 50 L/min, and that of AUX gas was 15 L/min.

The peak areas were directly chosen by ourselves in all data. We estimated the NPY carry-over as the ratio of the NPY mass chromatogram peak abundance for the blank to that of the last NPY analysis.

## RESULTS AND DISCUSSION

The analysis involved the injection of 1 μL aliquots a 1 μM NPY standard on an LC-Orbitrap MS instrument. Figure 1 shows the mass chromatograms with a mass window between *m*/*z* 712.84 to 712.87 (Fig. 1A–C). The protonated NPY [M+6H]^6+^ was the most abundant isotopic mass at *m*/*z* 712.8561 (Fig. 1D). The retention time was between 15.97 and 16.18 min using the Aeris PEPTIDE XB-C18 reversed phase column. We estimated the NPY carry-over as the ratio of the abundance of the NPY mass chromatogram peak for the blank to that of the most recent NPY analysis. The carry-over (%) for a 1 μL portion of 1 μM NPY was 4.05%, which was higher than the values for 1 μL of 5- and 10-μM (0.36, 0.47%, respectively) as shown in Table 1. The carry-overs of NPY of 5–10 μM were sufficiently low (0.36, 0.47%) and these were insignificant. Thus, in measurements of very small sample quantities such as biomolecules from real samples (there are less than a few pmol of NPY in a mouse brain) and in the case of the 1 μL–1 μM NPY standard, the carry-over effect is severe. This impinges on the accuracy of the quantitative analysis and it is essential to reduce this carry-over. The detection limit of the NPY standard in our LC-MS system was 0.05 pmol (1 μL of 0.05 μM). Our goal was, therefore, to reduce the carry-over (to less than 1%) in the 1 μL–1 μM NPY analysis.

(A) Mass chromatogram of LC-MS analysis of 1 μL–1 μM NPY. (B) Mass chromatogram of LC-MS analysis of the blank to estimate NPY carry-over. (C) Expanded mass chromatogram. (D) Mass spectrum of the analyzed NPY.

**Table 1:** Table 1. The carry-over ratios of the LC-MS analysis of NPY.

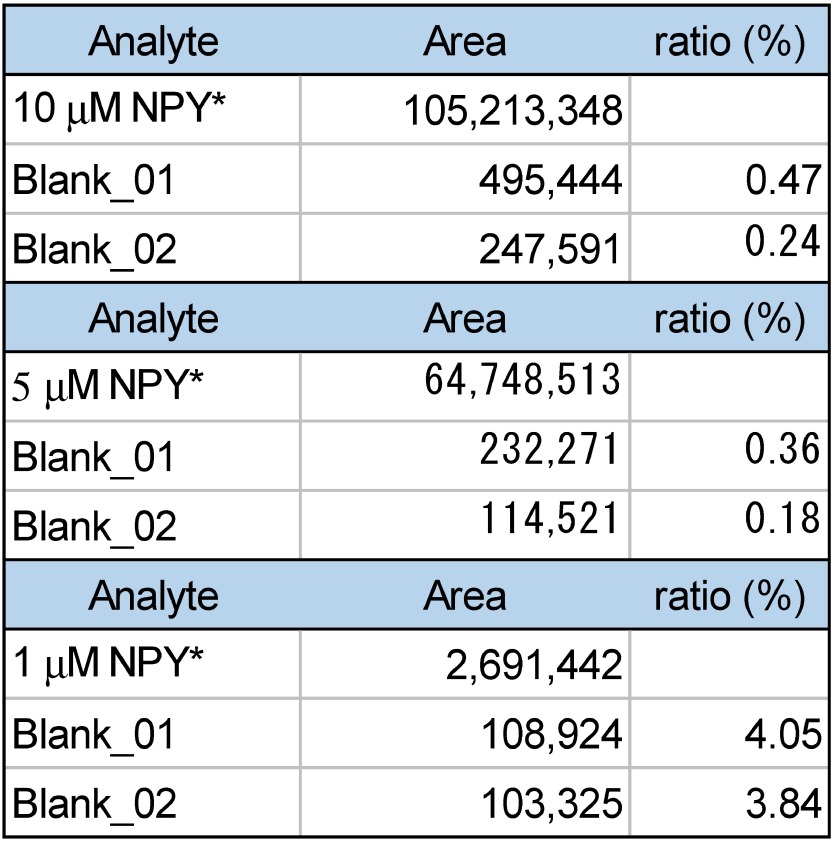

*The injection volume is 1 μL.

A simple way to reduce carry-over is to repeat the LC measurements of the blanks before the next experiment, however, the carry-over in four consecutive blank analyses was still significant after 1 μL–1 μM NPY measurement (1st and 2nd blank data shown in Table 1. 3rd and 4th blank data are not shown).

### Where are the candidate sites for carry-over located?

Generally speaking, the complete whole LC-MS system should be cleaned before starting experiments because contaminants in the LC-MS system could hamper analyte ionization and quantitative analysis, thus making it difficult to identify the origin of the contamination. The possible candidate sites for carry-over are locations where sample solution passes through. There is a high probability that carry-over could occur in the auto-sampler because the analytes are injected into the LC system using such a system. The candidate parts of the auto-sampler instrument are the sampling needle, the injection loop, lines and mechanical seals and valves. The column is also a good candidate for carry-over. In the MS instrument, the ion-source unit is usually contaminated by analytes and eluting materials. It is therefore important to clean the ion source unit including the cone, transfer tube and capillary tube in the ion probe prior to the LC-MS experiments. The candidate sites for carry-over are summarized in Table 2. We checked these possible candidate sites for their contribution to carry-over one by one.

**Table 2:** Table 2. Candidate sites of carry-over in LC-MS system.

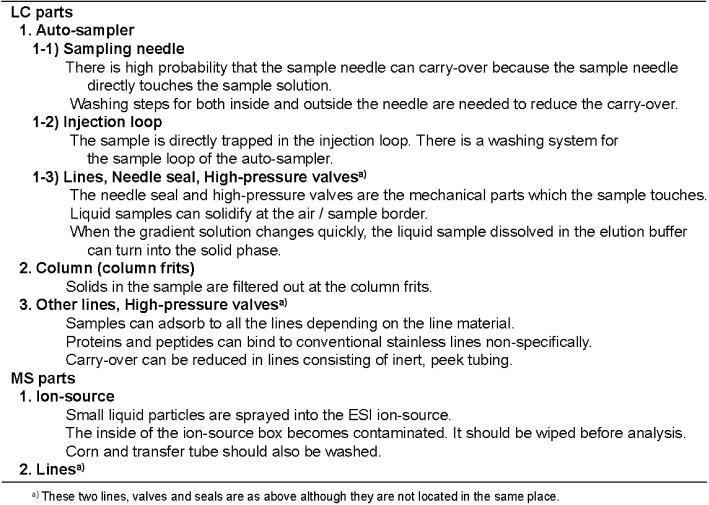

### Strategy for identifying carry-over sites

**Figure 2:**
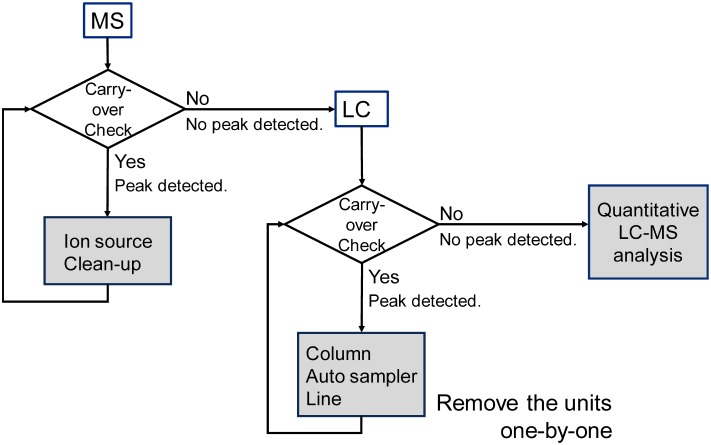
Fig. 2. Troubleshooting strategy for analyzing carry-over in LC-MS.

LC-MS analysis systems consist of LC and MS parts. To elucidate whether carry-over occurs in the LC or in the MS parts, carry-over at the MS instrument was initially checked using the solution from an LC pump or a syringe pump with the elution solution as a blank. If the carry-over peaks are detected in the eluting solution, then the carry-over occurs in the MS system. In such a case cleaning the ion source (cone, transfer tube, and capillary tube in the ion probe) would be needed. This is achieved by sonicating them in water/methanol or isopropanol. If the carry-over is not reduced, these items are replaced and the carry-over check repeated (Fig. 2).

If carry-over does not occur in the MS part then it must occur in the LC system. There are three possible locations where carry-over can occur, namely the auto-sampler, the column and the lines, as shown in Table 1. The three candidate parts need to be investigated independently.

We designed three experiments to determine the precise location where the carry-over could occur in the LC system. We estimated the extent of the NPY carry-over in these three different LC systems; 1 μL aliquots of 1 μM NPY were analyzed, after which, four consecutive blanks were analyzed. The data are summarized in Table 3.

**Table 3:** Table 3. The carry-over ratios in three experiments where LC parts were removed one-by-one.

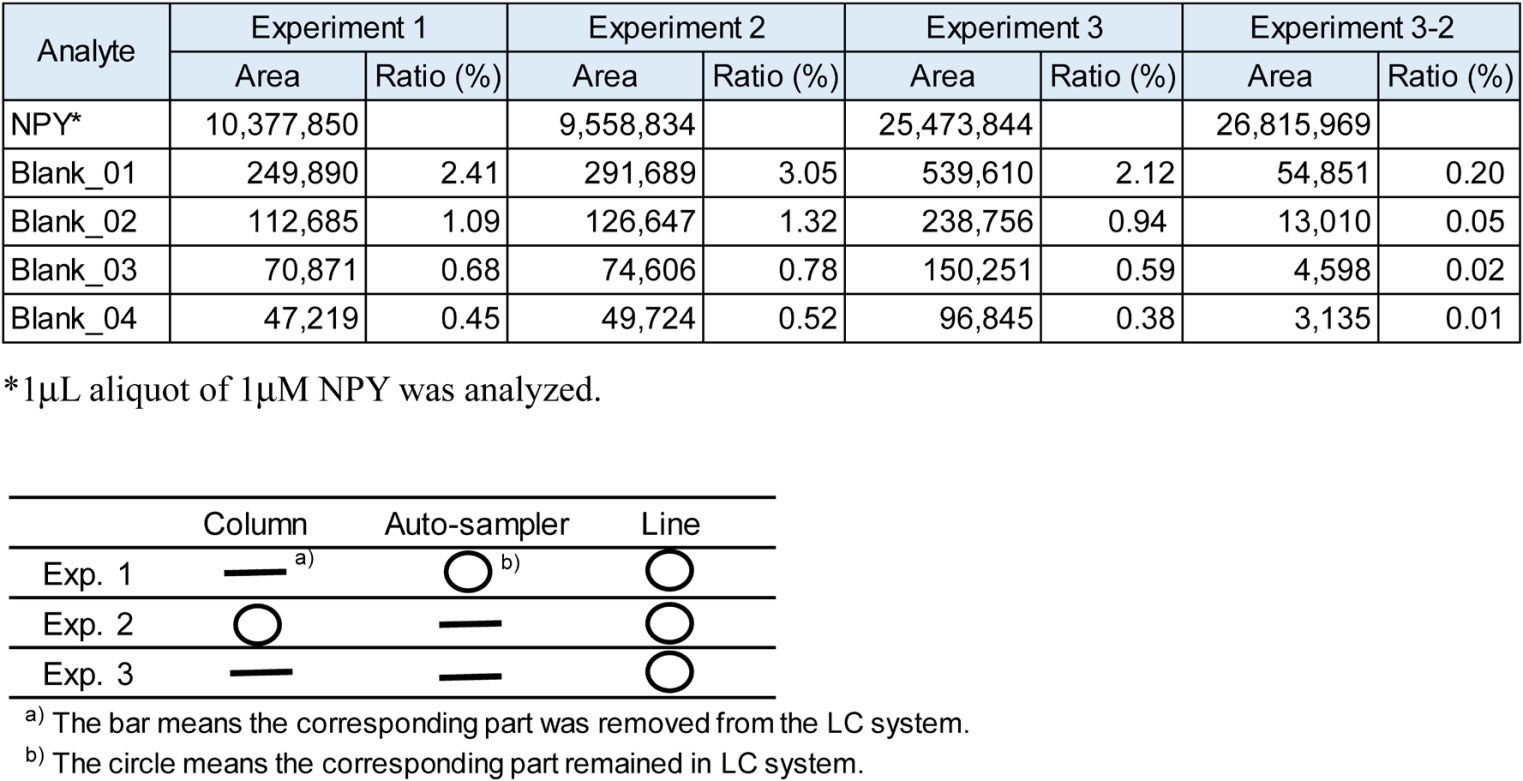

### Experiment 1: without column

The column was removed from the LC-MS system. The carry-over ratio for the first blank analysis was reduced from 4.05 to 2.41% in this system (Table 3). It can therefore be assumed that the carry-over of the column is large. After an NPY analysis, four blanks were measured sequentially.

**Figure 3:**
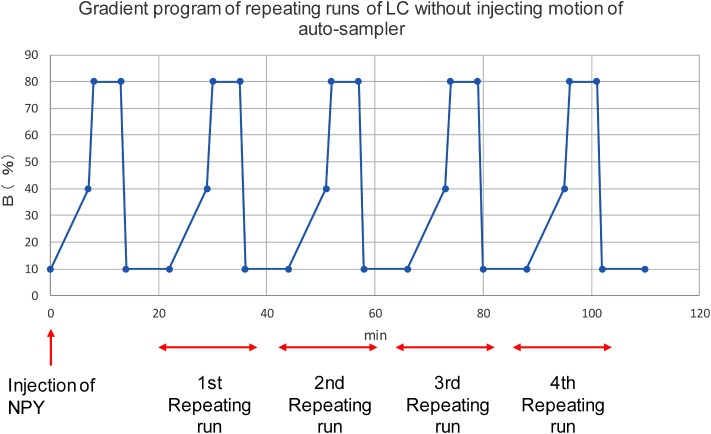
Fig. 3. Gradient program of repeated runs of LC without injecting motion of sample-needle in auto-sampler.

We also use a guard column and we compared the carry-over with or without the guard column (Table 4). The carry-over was reduced in the LC-MS system lacking a guard column (2.15%). The carry-over on the column system is particularly noticeable on the column frits. Therefore, we used an analytical column without a guard column in order to reduce the possible carry-over sites. However, the carry-over also remained when only the analytical column was used. For reducing column carry-over, it is necessary that the repeated blank analyses are performed as shown in Tables 3 and 4 or that the repeated cycles of the elution gradient program are added after NPY analysis, as shown in Fig. 3.

**Table 4:** Table 4. The carry-over ratio of the optimized LC-MS system.

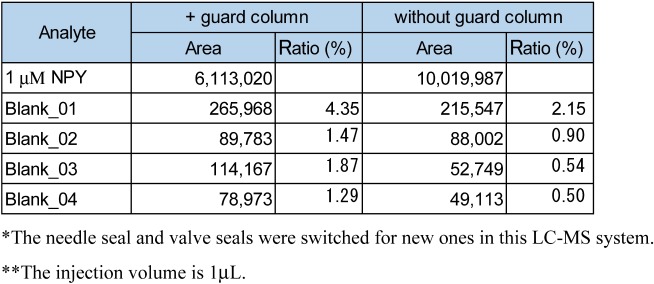

### Column

We compared a conventional C18 column (COSMOSIL 5C18-AR-II, Nakalai Tesq) with an Aeris PEPTIDE column which we mainly used. The carry-over of NPY with the COSMOSIL-column (1st blank 20.51%, 2nd blank 11.09%, 3rd blank 8.61% and 4th blank 6.64) were much more severe than the values for the Aeris PEPTIDE column, The COSMOSIL 5C18 column is designed to permit a broad spectrum of compounds such as small organic compounds, natural products, and small peptides to be analyzed. Thus, the concentration of the C18 chains on the resin surface is relatively high. In contrast, the Aeris PEPTIDE XB-C18 column is designed to specifically separate peptides, and the concentration of the C18 chains is less than that for the COSMOSIL column resin. Therefore, the carry-over of the NPY neuropeptide Y was more effectively reduced when the Aeris PEPTIDE column was used.

### Experiment 2: without the sample-needle of the auto-sampler

Four cycles in the elution gradient program were added after the NPY analysis as shown in Fig. 3. The sample needle was not used in the blank analysis. We can thus estimate the carry-over from the sample needle of the auto-sampler. It is thought that the sample needle of the auto-sampler can act as a source of carry over because it is in direct contact with the analyte. The carry-over was reduced to 3.05% from 4.05% (Tables 1 and 3). Thus, the auto-sampler is one of the source of NPY carry-over but it is not a major contributor. It is noteworthy that the carry-over in the repeated eluting gradient cycles was reduced with each elution. This gradient program is useful for reducing carry-over and for the practical analysis of NPY.

### Experiment 3: using the sample-needle of the auto-sampler without a column

In Experiment 3, the column was removed from the LC-MS and the LC gradient program used was the same as in Experiment 2. This allows the carry-over from the lines including the lines in the auto-sampler instrument to be estimated. The carry-over ratio difference was 2.12%. From all of the above findings we conclude that the carry-over from the column is in excess of 1% and that the sample needle of the auto-sampler contributes almost 1%. Unfortunately, the contribution of the lines including the lines and valves in the auto-sampler was the highest being nearly 2%.

To investigate this issue further we replaced the sample-needle seal and high-pressure valve seal which are consumable parts the auto-sampler. We then repeated Experiment 1 with new seals. The carry-over of NPY almost disappeared in the LC-MS lacking the column (Experiment 3-2 in Table 3). The carry-over of NPY was reduced to 2.15% at the first blank analysis and those of the second, third, and forth blank analyses were less than 0.9% in this LC-MS system (Table 4).

## CONCLUSION

We describe an approach for troubleshooting the carry-over of NPY in an LC-MS analysis. Although the carry-over has the potential to occur anywhere and the situation is complex, considering and checking where they occur is a worthwhile endeavor. We describe a strategy for determining where the carry-over occurs by removing candidate parts in the LC-MS system one-by-one. The findings reported in this study indicate that the carry-over of NPY occurs from the column, the guard column in particular and at the consumable seals of sample-needle and high-pressure valves.

## Notes

Mass Spectrom (Tokyo) 2019; 8(2): S0083
